# The Elg1-RFC Clamp-Loading Complex Performs a Role in Sister Chromatid Cohesion

**DOI:** 10.1371/journal.pone.0004707

**Published:** 2009-03-05

**Authors:** Marie E. Maradeo, Robert V. Skibbens

**Affiliations:** Department of Biological Sciences, Lehigh University, Bethlehem, Pennsylvania, United States of America; University of Edinburgh, United Kingdom

## Abstract

It is widely accepted that of the four Replication Factor C (RFC) complexes (defined by the associations of either Rfc1p, Ctf18p, Elg1p or Rad24p with Rfc2p-Rfc5p), only Ctf18-RFC functions in sister chromatid cohesion. This model is based on findings that *CTF18* deletion is lethal in combination with mutations in either *CTF7^ECO1^* or *MCD1* sister chromatid cohesion genes and that *ctf18* mutant cells exhibit cohesion defects. Here, we report that Elg1-RFC not only participates in cohesion but performs a function that is distinct from that of Ctf18-RFC. The results show that deletion of *ELG1* rescues both *ctf7^eco1^* mutant cell temperature sensitivity and cohesion defects. Moreover, over-expression of *ELG1* enhances *ctf7^eco1^* mutant cell phenotypes. These findings suggest that the balance of Ctf7p^Eco1p^ activity depends on both Ctf18-RFC and Elg1-RFC. We also report that *ELG1* deletion produces cohesion defects and intensifies the conditional phenotype of *mcd1* mutant cells, further supporting a role for Elg1-RFC in cohesion. Attesting to the specificity of these interactions, deletion of *RAD24* neither suppressed nor exacerbated cohesion defects in either *ctf7^eco1^* or *mcd1* mutant cells. While parallel analyses failed to uncover a similar role in cohesion for Rad24-RFC, it is well known that Rad24-RFC, Elg1-RFC and Ctf18-RFC play key roles in DNA damage responses. We tested and found that Ctf7p^Eco1p^ plays a significant role in Rad24-RFC-based DNA response pathways. In combination, these findings challenge current views and document new and distinct roles for RFC complexes in cohesion and for Ctf7p^Eco1p^ in DNA repair.

## Introduction

Sliding clamps participate in numerous facets of DNA metabolism that include DNA replication, DNA repair and chromatin modifications such as assembly of higher-order chromatin complexes [1]. Inherent to their function, sliding clamps such as the homotrimeric PCNA (encoded by *POL30* in budding yeast) or heterotrimeric Rad17p-Mec3p-Dcd1p alternate clamp form a topologically-closed ring-like structure that encircles DNA. These sliding clamps remain stably attached to chromatin but also are able to move along the chromosome length – providing a mobile landing platform from which replication, repair and modifying factors can access DNA.

Not surprisingly, topologically closed sliding clamps require special factors for loading onto DNA and also for subsequent unloading. A family of Replication factor C (RFC) complexes use multiple ATP hydrolysis reactions to open topologically closed sliding clamps and provide for clamp loading/unloading onto DNA [2], [3]. Rfc1-RFC is the only essential RFC complex and opens PCNA rings for loading onto RNA-primed DNA junctions during DNA replication or DNA repair. Ctf18-RFC also catalyzes PCNA loading/unloading, but *CTF18* is not essential for cell viability [4]–[11]. A physical interaction between Elg1p and PCNA suggest that Elg1-RFC may also open and thus load PCNA onto DNA, however, direct evidence for such a role remains to be documented [12]. Rad24-RFC is unique in that it opens the Rad17p-Mec3p-Ddc1p alternate sliding clamp for loading onto DNA and appears dedicated to DNA repair functions [13], [14]. However, Rad24-RFC also participates in PCNA opening during unloading reactions [3].

While the functions of various RFC complexes (and their cognate sliding clamps) in DNA replication and repair are well established, much less is known about their roles in chromatin modification. The most intriguing example for chromatin modification is provided by Ctf18-RFC – whose function is critical to establish sister chromatid pairing during S-phase [10], [11], [15]. Appropriate cohesion involves identifying the products of chromosome replication as sisters, depositing cohesins onto each sister and then modifying those cohesins to form structural bridges that tether together sister chromatids until anaphase onset [16], [17]. Evidence that RFCs and the PCNA sliding clamp function in cohesion originated from observations that yeast cells containing mutations in either *CTF18* or *POL30* are lethal when combined with mutations in *CTF7/ECO1* [15]. Ctf7p^Eco1p^ is an acetyltransferase that activates cohesins during S-phase and is essential for sister chromatid pairing [15], [18]–[21]. Loss of *CTF18* is also lethal in combination with mutations in *MCD1*, which encodes a structural subunit of the cohesin complex [22], [23]. Subsequent studies revealed that both *ctf18* and *pol30* mutant cells exhibit precocious sister chromatid separation [10], [11], [24]. Based on these findings and also that Ctf18-RFC participates in PCNA dynamics, PCNA cohesion activity was postulated early on to occur through interactions with Ctf18-RFC [24]–[26].

The specific and unique function of Ctf18-RFC in cohesion, to the exclusion of other PCNA-loading complexes such as Rfc1-RFC and Elg1-RFC, is prevalent in the literature [1], [5], [27]. In contrast to this widely accepted view, alternative RFCs often compensate for one another during DNA replication checkpoint activation and repair [5], [28]. Prior findings indicate that each RFC complex associates in vitro with Ctf7p^Eco1p^ [29, B. Satish and R. V. Skibbens, unpublished], suggesting that Ctf18-RFC may not be unique in its cohesion function. Moreover, a large body of evidence obtained from high through-put screens identified genetic interactions between *ELG1* and *RAD24* with each other, as well as with *RFC1* and *CTF18*, and also with numerous cohesion-related genes that include *CHL1*, *CTF4*, *POL30* and small *RFC* subunits [5], [23], [27]–[34,]. Here, we pursue these findings and provide new evidence that Elg1-RFC plays a key role in cohesion establishment. Our results demonstrate that Elg1p exhibits cohesion activities and that this activity is distinct from that exhibited by Ctf18p. Our findings also reveal interactions between *RAD24* and both *CTF7^ECO1^* (cohesion establishment) and *MCD1* (cohesion maintenance). Finally, we provide new evidence that Ctf7p^Eco1p^ participates in Rad24-RFC dependent DNA repair pathways. As such, these findings suggest the need for a shift in current thinking regarding RFC function in cohesion.

## Results

### *ELG1* deletion, but not *RAD24* deletion, suppresses *ctf7* mutant cell defects

**Figure 1:**
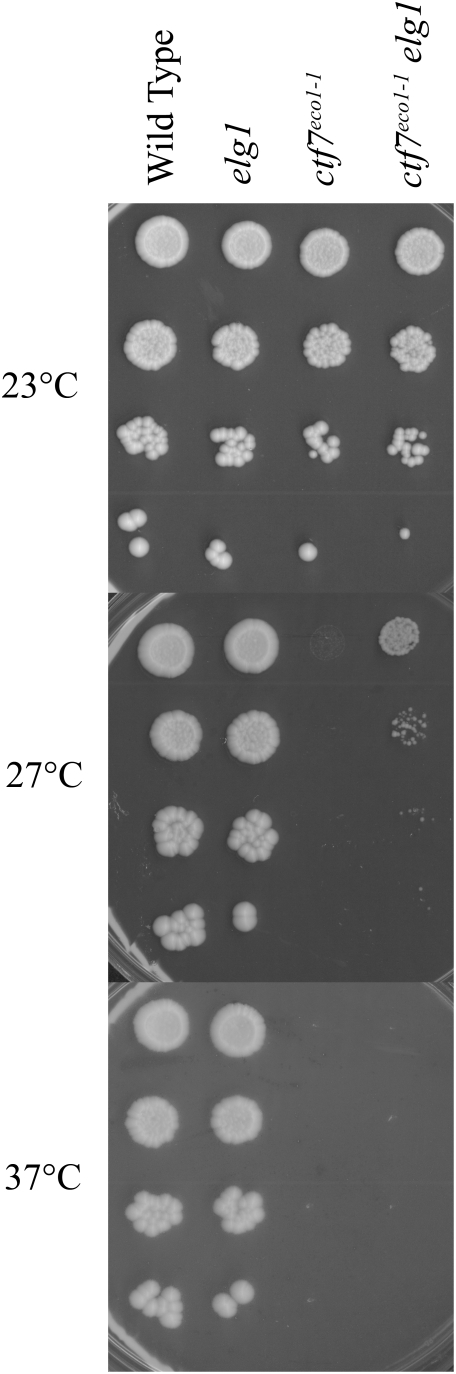
*ELG1* deletion suppresses *ctf7* mutant cell conditional growth. 10-fold serial dilutions of wildtype, *ctf7* and *elg1* single mutant strains compared to *ctf7 elg1* double mutant strains. Colony growth shown for cells on rich medium plates maintained at 23°, 27° and 37° for 7 days.

**Figure 2:**
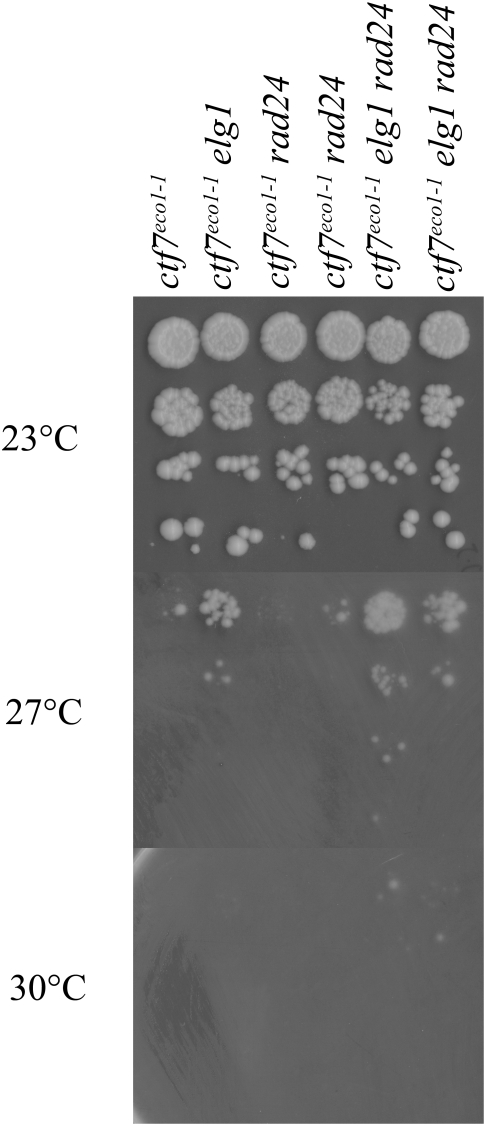
*RAD24* deletion neither rescues nor exacerbates *ctf7* mutant cell conditional growth. 10-fold serial dilutions of *ctf7* single mutant strains compared to *ctf7 elg1* and *ctf7 rad24* double mutant strains as well as to *ctf7 elg1 rad24* triple mutant cells. Colony growth shown for cells on rich medium plates maintained at 23°, 27° and 30° for 7 days.

Based on findings that RFCs often exhibit functional redundancy and that all RFC subunits associate with Ctf7p in vitro [5], [28], [29, Satish and Skibbens, unpublished data], we tested whether either Elg1p or Rad24p might participate in cohesion establishment. Site-directed PCR mutagenesis was used to replace either the full length *ELG1* or *RAD24* open reading frame with a selectable marker and the appropriate knock-outs confirmed by PCR. The resulting mutant strains were then crossed into a *ctf7^eco1-1^* yeast strain conditional for cohesion establishment, sporulated and the resulting double mutant haploid cells identified. Cells harboring combinations of mutated *ctf7* with either *elg1* or *rad24* deletions are viable. Additional crosses between these resulting strains produced viable cells that harbored all three alleles (*ctf7*, *elg1* and *rad24*). While these findings at first appear to support the simple and popular model that only Ctf18-RFC functions in cohesion and that alternate RFC complexes are not functionally redundant in this respect, these and prior efforts fail to take into account that RFC complexes might have distinct roles in cohesion establishment. To test this possibility, 10-fold serial dilutions of each single (*ctf7*, *elg1* or *rad24*) and double mutant strain (*ctf7 elg1* and *ctf7 rad24*) were grown at a range of temperatures. As expected, wildtype cells grew at all temperatures tested while *ctf7* mutant cells were inviable at temperatures that exceeded 27°. Surprisingly, loss of *ELG1* rescued *ctf7*-dependent conditional growth such that *ctf7 elg1* cells remained viable at temperatures lethal for *ctf7* single mutant cells (Figure 1). To confirm this result, we deleted *ELG1* in an independent *ctf7* allele (*ctf7-203*). The results show that *ctf7-203 elg1* double mutant cells also remain viable at temperatures lethal for *ctf7* single mutant cells (Figure S1). In contrast, loss of *RAD24* failed to either rescue or exacerbate the conditional growth phenotype in either a *ctf7^eco1-1^* or *ctf7-203* mutant background (Figure 2, data not shown).

We considered the possibility that Rad24-RFC might play a cryptic role in cohesion that could be uncovered by deleting *RAD24* in the context of a *ctf7 elg1* double mutant background. In testing this possibility, we generated several *ctf7 elg1 rad24* triple mutant isolate strains and found that each exhibited growth characteristics similar to those of *ctf7 elg1* double mutant cells (Figure 2). Occasionally, a *ctf7 elg1 rad24* isolate appeared to marginally enhance growth over *ctf7 elg1* double mutant (one such isolate is shown in Figure 2). However, a growth advantage was not observed in *ctf7-203 elg1 rad24* triple mutant cells, relative to *ctf7-203 elg1* double mutant cells (data not shown). In combination, these results suggest that Elg1p participates in cohesion and that Elg1-RFC does not function redundantly to Ctf18-RFC. In addition, a third RFC complex (Rad24-RFC) fails to significantly influence Elg1-RFC cohesion establishment pathways.

### Elg1p is a cohesion factor that, upon deletion, reduces *ctf7* mutant cohesion establishment defects

**Figure 3:**
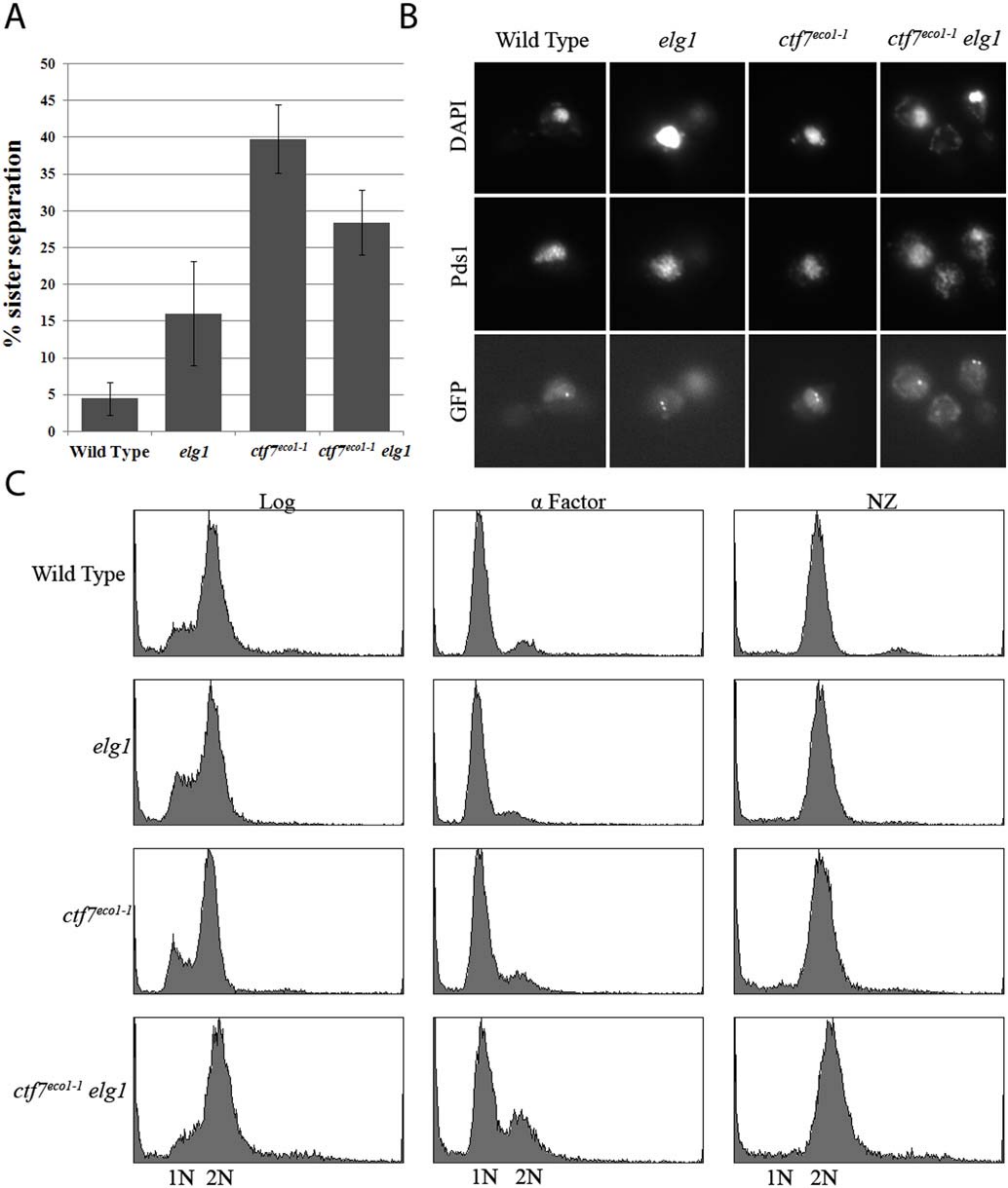
Role of *ELG1* deletion in cohesion. A) Quantification of cohesion defects exhibited by wild type, *ctf7* and *elg1* single mutant strains and *ctf7 elg1* double mutant strains arrested prior to anaphase. Error bars represent standard deviation. B) Micrographs of wild type, *ctf7* and *elg1* single mutant strains and *ctf7 elg1* double mutant strains in which sister chromatid loci (GFP) and Pds1p (Pds1) are visualized within the DNA mass (DAPI). C) DNA profiles of wild type, *ctf7* and *elg1* single mutant strains and *ctf7 elg1* double mutant strains during log phase growth (Log), synchronized in G_1_ (α-Factor) at 23°C and then released into 30°C fresh medium containing nocodazole (NZ) to arrest cells prior to anaphase.

That loss of Elg1p suppresses the conditional lethality of *ctf7* mutant cells prompted us to test whether the physiological basis for this rescue is due to diminished cohesion defects normally associated with *ctf7* mutant cells [15], [18], [24]. To address this issue, wildtype, *ctf7* single mutation, *elg1* single mutation and *ctf7 elg1* double mutations were crossed into a cohesion assay strain in which *TetO* arrays are integrated approximately 40 kb from centromere V and detected via constitutive expression of GFP-tagged *TetR-GFP TetO*-binding protein [10], [35]. This cohesion assay strain also contains epitope-tagged Pds1p, allowing for detection of this inhibitor of anaphase onset [36]. Sporulation produced isolates of each strain in which the location of both sister chromatid loci and retention of Pds1p could be monitored by microscopy. The resulting wildtype, *ctf7* single mutant cells, *elg1* single mutant cells and *ctf7 elg1* double mutant cells were incubated at permissive temperature (23°C) in fresh medium supplemented with alpha factor for three hours to synchronize the cultures in the G_1_ portion of the cell cycle. Repetitive analyses revealed that *ctf7 elg1* double mutant cells failed to synchronize in G_1_ with the same efficiency as either *ctf7* or *elg1* single mutant strains. Only replicates in which *ctf7^eco1-1^ elg1* double mutants exhibited a robust G_1_ arrest were used in subsequent cohesion analyses. In addition to this method, we decided to include a separate strategy in which log phase cells were synchronized in early S-phase using medium supplemented with hydroxyurea (HU), which produced a more effective synchronization (data not shown). For both synchronization strategies, each culture was washed and then shifted to a semi-permissive temperature (see Materials and Methods) in fresh medium supplemented with nocodazole to synchronize the cultures in the M-phase portion of the cell cycle. Pre-anaphase M-phase arrested cells were confirmed by the presence of a 2C DNA content by flow cytometry, large-budded cell morphology via microscopy, and retention of Pds1p content via epi-fluorescence (see Materials and Methods). From these cells, we then ascertained the disposition of GFP-labeled sister chromatid loci. As expected, pre-anaphase wildtype cells arrested as large budded cells that contained Pds1p co-incident with DAPI staining and closely apposed sister chromatids such that few (4%) of GFP-labeled sister chromatids were separated (Figure 3). While pre-anaphase *ctf7* mutant cells similarly arrested as large budded cells with Pds1p co-incident with DAPI staining, these cells exhibited a robust cohesion defect such that roughly 40% of the cells contained separated sister chromatids. The essential role for Ctf7p in cohesion has been previously reported [15], [18]. Importantly, deletion of *ELG1* significantly reduced the incidence of separated sister chromatids in the *ctf7* mutant background – regardless of the pre-synchronization strategy employed (Figure 3). The finding that loss of *ELG1* decreases the precocious sister separation defect in *ctf7* mutant cells is consistent with our data that *elg1* deletion partially recues *ctf7* temperature sensitivity and in a pathway separate from Ctf18p function.

If Elg1p affects the balance of Ctf7p function, relative to Ctf18p, then deletion of *ELG1* might be sufficient to produce a cohesion defect. We extended the above analyses to include both wildtype and *elg1* deletion mutant strains in the cohesion assay background and found that approximately 15% of large-budded *elg1* mutant cells that retain Pds1p staining also contain separated sister chromatids (Figure 3). To confirm this result, log phase wildtype and *elg1* mutant cells were placed into fresh 23° medium supplemented nocodazole for 3 hours to obtain mitotic pre-anaphase arrest. As expected, pre-anaphase wildtype cells contained tightly paired sister chromatids such that very few (6%) sisters were dissociated. In contrast, *elg1* mutant cells arrested in pre-anaphase contained a significantly higher level (20%) of precociously separated sister chromatids (Figure S2) – despite the retention of Pds1p in these cells. Wild type and *elg1* cells arrested in α factor for 3 hours at 23°C both contained 4% or less dissociated sister chromatids. Similarly, *elg1* arrested in early S-phase using HU also contained 2% dissociated sisters. Thus, the increased incidence of 2 GFP signals in *elg1* cells is due to precocious sister separation and not to pre-existing aneuploidy or gross chromosomal rearrangements (Figure S2). This *ELG1* deletion-dependent cohesion defect was consistently recapitulated in numerous assays and is consistent with levels reported in numerous other DNA replication/repair-related mutant strains [10], [11], [24], [29], [30], [37]–[39].

### *ELG1* over-expression enhances *ctf7* mutant cell growth defects

**Figure 4:**
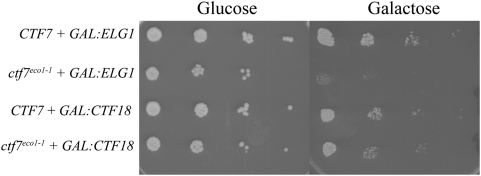
*ELG1* over-expression reduces *ctf7* mutant cell growth. 10-fold serial dilutions of wildtype and *ctf7* mutant cells harboring plasmid from which either *ELG1* or *CTF18* expression is repressed (Glucose) or elevated (Galactose).

The finding that *ELG1* deletion suppresses *ctf7* mutant cell temperature sensitivity suggests that *ELG1* over-expression might in turn exacerbate *ctf7* mutant cell conditional phenotypes. To test this predication, wild type and *ctf7^eco1-1^* cells were transformed with a plasmid in which either *ELG1* or *CTF18* expression from the *GAL1-10* promoter could be induced to high levels upon exposure to medium containing galactose [7]. 10-fold serial dilutions of the resulting transformed cells at log phase growth were plated on media containing either galactose (elevate *ELG1* or *CTF18* expression) or glucose (repress *ELG1* or *CTF18* expression) and maintained at 23°C. The results show that *ELG1* overexpression significantly intensified the conditional phenotype of *ctf7* mutant cells at 23°C but had no effect in wild type cells (Figure 4). This enhanced conditional phenotype is *ELG1*-dependent given that both wildtype and *ctf7* mutant cells devoid of plasmid exhibited equivalent growth when plated on galactose (data not shown). In contrast, overexpression of *CTF18* neither suppressed nor enhanced the conditional phenotype of *ctf7* mutant cells. In combination, these findings reveal that Ctf7p-dependent cohesion establishment activity is highly sensitive to Elg1-RFC dosage and in a fashion that is separate from that of Ctf18-RFC dosage.

### ***ELG1* promotes sister chromatid cohesion**

**Figure 5:**
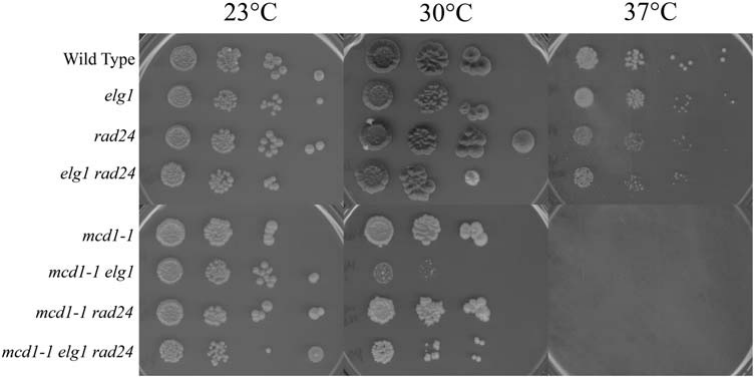
*ELG1* deletion, but not *RAD24* deletion, exacerbates *mcd1-1* mutant cell conditional growth. 10-fold dilutions for each single, double and triple mutant strain are shown (see text). Colony growth shown on rich medium plates maintained at 23°, 30° and 37° for 7 days.

*CTF18* deletion is lethal in combination with either *ctf7* cohesion establishment or *mcd1* cohesion maintenance alleles [15], [23]. In contrast, *ELG1* deletion suppresses both *ctf7*-dependent temperature sensitivity and cohesion establishment defects (this study). Thus, it became important to test whether *ELG1* deletion would rescue or exacerbate *mcd1*-dependent conditional growth. Single mutant *elg1* and *mcd1-1* strains were crossed and sporulated to obtain *mcd1-1 elg1* double mutant cells. 10-fold serial dilutions of log phase cells were then plated onto rich medium and incubated at a range of temperatures. In contrast to the suppression of *ctf7* phenotypes provided by *ELG1* deletion (Figure 2), conditional growth of mutant *mcd1-1* cells was significantly exacerbated by *ELG1* deletion (Figure 5). This finding reveals a novel interaction that differentiates Elg1-RFC-functions in cohesion establishment and maintenance pathways.

We next asked whether *RAD24* deletion might similarly affect the cohesion maintenance pathway. To address this, strains harboring the cohesin mutant *mcd1-1* were crossed to a strain harboring *elg1 rad24* double deletions, sporulated and isolates of the appropriate wildtype, *elg1*, *rad24* and *mcd1-1* single mutant strains, *elg1 rad24*, *mcd1-1 elg1* and *mcd1-1 rad24* double mutant strains and *mcd1-1 elg1 rad24* triple mutant strains identified. We then tested these against a range of growth temperatures. The results show that *RAD24* deletion neither positively nor adversely affected the conditional growth phenotypes associated with the *mcd1-1* cohesin mutant strain (Figure 5). Surprisingly, *rad24 mcd1-1 elg1* triple mutant cells exhibited an increase in growth compared to *mcd1-1 elg1* double mutant cells (Figure 5). These observations raise the possibility that either cohesin defects associated with diminished Mcd1p function contributes to inappropriate DNA damage responses that act through Rad24-RFC or that Rad24-RFC normally inhibits cohesin pathways and thus partially rescues growth defect in *mcd1-1 elg1* mutant cells.

### Role of Ctf7p in RFC-dependent responses to DNA damage

**Figure 6:**
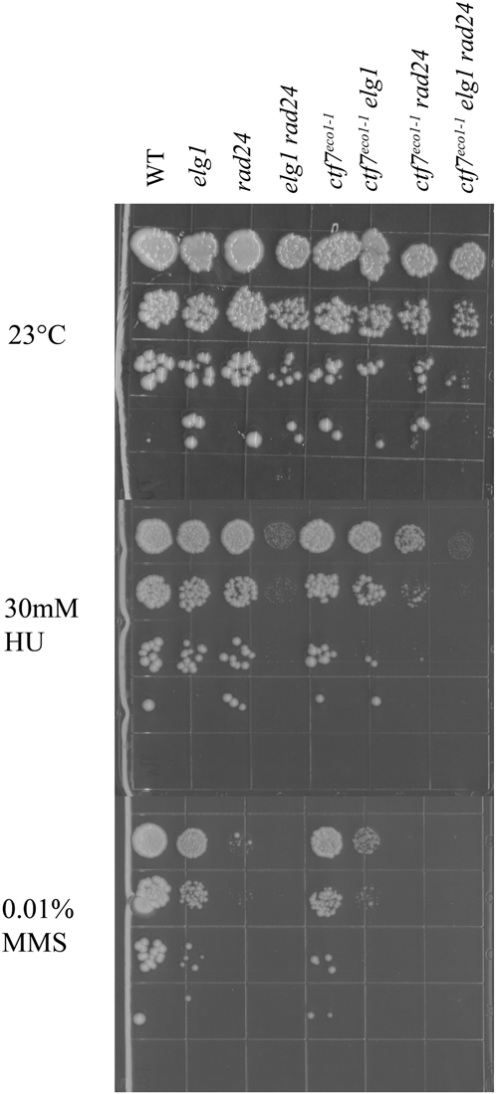
Ctf7p function in DNA repair. 10-fold serial dilutions of *ctf7*, *elg1* and *rad24* single mutant strains, *elg1 rad24*, *ctf7 elg1*, *ctf7 rad24* double mutant strains and *ctf7 elg1 rad24* triple mutant strains grown at 23° on rich medium and medium supplemented to either 30 mM hydroxyurea (HU) or 0.01% methyl methyl sulfanate (MMS).

*ctf18*, *elg1* and *rad24 single* mutant strains exhibit hypersensitivities to genotoxic agents such as methyl methane sulfonate (MMS) or hydroxyurea (HU) [5], [28]. There is a single report that *ctf7* mutant cells exhibit sensitivity to double-strand breaks induced by γ-irradiation – but the basis for this effect was posited as indirect (mediated through loss of sister templates) [40], [41]. However, recent evidence revealed that Ctf7p function is up-regulated in G_2_/M by double strand breaks to induce additional cohesion establishment activities [42], [43]. Thus, we asked whether we could reveal a new DNA damage response role for Ctf7p in RFC mutant strains sensitized to DNA damage. Serial dilutions of each single (*elg1*, *rad24* and *ctf7*), double and triple mutant strain were plated onto 23° rich medium plates and also media plates supplemented with either DNA alkylating reagent (MMS) or replication fork-stalling reagent (HU). In response to MMS, both *rad24* and *elg1* exhibited decreased growth with *rad24* single mutant cells exhibiting the more dramatic adverse effect. *elg1 rad24* double mutant cells were inviable in response to MMS exposure – consistent with the functional redundancies of these alternate RFC complexes [5], [28]. At best, *ctf7* mutant cells exhibited a very modest growth defect in response to MMS. In contrast, both *egl1 ctf7* and *rad24 ctf7* double mutant cells exhibited synergistic growth defects in response to MMS (Figure 6). The role for Ctf7p in DNA damage response is recapitulated by testing for growth in response to hydroxyurea. *elg1*, *rad24* and *ctf7* single mutant cells all exhibited relatively little growth defects upon exposure to hydroxyurea. In contrast, *elg1 rad24* double mutant cells were severely growth limited – again attesting to the redundancies of these RFC complexes. Similarly *elg1 ctf7* and *rad24 ctf7* double mutant cells both exhibited increased sensitivity to HU exposure, relative to the single mutant strains, with *rad24 ctf7* providing the more dramatic growth defect (Figure 6). In combination, these studies reveal for the first time that Ctf7p performs a key role in response to both replication fork stalling and alkylation-dependent DNA damage response pathways.

## Discussion

Of the four unique RFC complexes, only Ctf18-RFC (and its cognate sliding clamp PCNA) have been linked to sister chromatid cohesion [5], [6], [10], [11]. Here, we provide multiple levels of new evidence that Elg1-RFC functions in sister chromatid cohesion and in a manner that is not redundant to Ctf18-RFC. First, *ELG1* deletion suppresses both the conditional growth and cohesion defect phenotypes exhibited by *ctf7* cohesion establishment mutant strains. Second, *ELG1* overexpression greatly exacerbates the conditional growth defect exhibited by *ctf7* mutant strains. In combination, these results reveal that Ctf7p cohesion function is greatly influenced by Elg1-RFC levels. In addition, *ELG1* deletion alone is sufficient to generate precocious sister chromatid separation and at levels consistent with numerous other DNA metabolism genes that exhibit dual roles in cohesion [17]. In support of our findings that Elg1-RFC functions in cohesion, previous studies showed that all RFC complexes physically interact with Ctf7p in vitro [29, B. Satish and R. V. Skibbens, unpublished data]. In addition, high through-put screens documented numerous genetic interactions between *ELG1* and gene products that function in cohesion [5], [23], [31], [32]. Finally, Rfc1-RFC, Ctf18-RFC and Elg1-RFC all associate with PCNA and PCNA itself promotes efficient cohesion establishment – indirectly linking Elg1-RFC to cohesion [1], [7], [15], [24]. Based on our current study, it is likely that Rfc1-RFC – and any other PCNA clamp-loading complex yet to be identified - will similarly participate in cohesion. Intriguingly, cells with mutations in PCNA produce only modest cohesion defects while cells with mutations in Ctf18p exhibit high levels of premature separation [10], [11], [26]. Our current findings provide some rationale for this observation in that reduced PCNA function may provide a balanced reduction of both Ctf18-RFC and Elg1-RFC activities which may act in an antagonistic fashion. However, this is likely to be an oversimplification of an extremely complex process.

**Figure 7:**
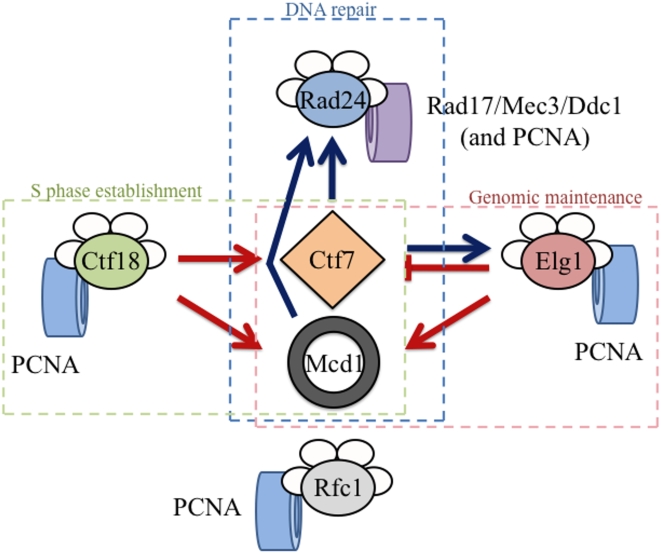
Schematic highlighting RFC interactions. Ctf18-RFC and Elg1-RFC exhibit separate and distinct activities relative to Ctf7p-dependent cohesion establishment but both promote Mcd1p-dependent cohesion maintenance. Ctf7p and Mcd1p both promote the Rad24-RFC DNA damage response pathway. Note that Ctf18-RFC, Elg1-RFC, Rfc1-RFC and Rad24-RFC all participate in PCNA clamp dynamics while Rad24-RFC functions exclusively in Rad17/Mec3/Ddc1 clamp dynamics. Cohesion-based pathways are shown in red, DNA damage repair pathways are shown in blue. Dashed boxes indicate RFC cellular roles.

The current study provides for at least two broadly defined mechanisms regarding the function of RFC complexes in cohesion: an “opposing activities” model and a “competing interaction” model (Figure 7). In the first model, Elg1-RFC and Ctf18-RFC exhibit opposing activities during cohesion establishment. Here, Ctf18-RFC might promote cohesion establishment through recruiting Ctf7p to chromatin or activating Ctf7p after recruitment. Elg1-RFC would then inhibit or reverse these recruitment/activation steps, possibly to limit cohesion establishment to S-phase when Ctf7p functions in unperturbed cells [15], [18]. All RFC complexes associate with Ctf7p in vitro, providing initial support for RFC-dependent Ctf7p recruitment to chromatin [29, Satish and Skibbens, unpublished data]. However, RFC complex recruitment of Ctf7p might instead occur indirectly, given that Ctf7p also binds PCNA [24]. In a second scenario, Ctf18-RFC and Elg1-RFC might exhibit opposing activities in PCNA loading and unloading, respectively. One complication to this scenario is that there is no direct evidence that Elg1-RFC functions in PCNA ring opening reactions. If the above model proves correct, then our study provides key functional data that Elg1-RFC functions in PCNA reactions and likely in direct opposition to Ctf18-RFC. We note that previous studies in *S. pombe* showed that the synthetic lethality of *rfc1 ctf18* double mutants was suppressed by the additional deletion of *elg1* – leading those authors to first propose that opposing Ctf18-RFC and Elg1-RFC activities were based on PCNA loading versus unloading reactions [44]. Our study extends this model to now include cohesion establishment reactions. However, we note that the inviability of *ctf7 ctf18 elg1* triple mutant reveals a more complicated scenario since the absence of Elg1p is not sufficient to counteract the lack of Ctf18p in cells diminished for Ctf7p activity (M. Maradeo and R. V. Skibbens, unpublished data).

The “competing interaction” model is predicated on, but not limited to, the physical interactions between Ctf7p and RFC complexes but posits that these RFC complexes do not necessarily act in direct opposition to one another. One of many formulations of this model is that Ctf18-RFC associates with Ctf7p during cohesion establishment while Elg1-RFC might bind Ctf7p during genomic maintenance/DNA repair. Here, loss of Elg1-RFC allows for more efficient binding of Ctf18-RFC to ctf7p mutant protein - providing for more efficient cohesion establishment activity. This model is supported by evidence that *elg1* deletion exacerbates *mcd1* mutant phenotypes, indicating that Elg1p function is critical for the ultimate maintenance of sister chromatin pairing. Relevant to this model is that *CTF7* exhibits numerous genetic interactions with *POL30* (PCNA), and that these interactions appear dependent on PCNA modifications [15], [24]. For instance, PCNA can be mono-ubiquitinated, poly-ubiquitinated or SUMOyated and these modifications occur during DNA replication or in response to genotoxic challenges [45]. Recent evidence revealed that Ctf7p activity is up-regulated in response to DNA damage so that sister chromatid pairing reactions can occur outside of S-phase and separate from DNA replication/repair forks [42], [43]. Elg1-RFC also functions during DNA damage and appears to suppress inappropriate recombinations [6], [12], [46], [47]. Based on this example, various RFC complexes may compete for Ctf7p binding such that changes in the dosage (deletion versus over-expression) can tip the balance of Ctf7p function.

Finally, the current study also provides new information regarding the role of Ctf7p in DNA repair. The results show that Ctf7p plays a critical role in responding to DNA damage induced by either alkylation-based bulky adducts or to stalled replication forks resulting from exposure to hydroxyurea. These effects are most significant when *ctf7* mutations occur in a *rad24* deletion background. As opposed to the three other RFC complexes, Rad24-RFC loads the heterotrimeric clamp composed of Rad17p, Mec3p and Ddc1p [14]. Our results that *ctf7 rad24* double mutant cells are severely growth compromised upon either MMS or HU exposure suggest the possibilities that Ctf7p functions in DNA damage responses by associating with the Rad17p-Mec3p-Ddc1p alternate sliding clamp or by stimulating Rad24-RFC activity. A third possibility is that Ctf7p functions in a DNA damage response pathway that is parallel to Rad24p [29].

In summary, the current study provides new evidence that replication fork components are critical for establishing sister chromatid cohesion and complicate the simple notion that RFC proteins have predominantly compensatory cellular roles. In addition, our results necessitate a shift in current perceptions of alternative RFC protein functions in cohesion.

## Materials and Methods

### Yeast strains and media

All strains used in this study were performed in the W303 background except for cells containing the *ctf7-203* allele – which are carried in the S288C background (see Table 1). To construct *CEN*-proximal cohesion assay strains containing *ctf7^eco1-1^:ADE*, plasmid containing wild type *CTF7* was genetically engineered to contain *ctf7^eco1-1^* DNA that harbors G211D. The *ADE2* gene was then inserted behind the *ctf7^eco1-1^* open reading frame but within the non-coding 3′ untranslated region of *CTF7*. The resulting fragment was transformed into the *CEN*-proximal cohesion assay strain.

**Table 1:** All strains are of the W303 background except strains labeled with * which are S288C background.

Strain	Genotype
YMM334	*MATa ade2-1 his3-11,15 leu2-3,112 trp1-1 ura3-1 CTF7:ADE2 URA3:tetO LEU2:tetR-GFP TRP1:PDS1-MYC13 (isolate 1)*
YMM335	*MATa ade2-1 his3-11,15 leu2-3,112 trp1-1 ura3-1 ctf7^eco1-1^:ADE2 URA3:tetO LEU2:tetR-GFP TRP1:PDS1-MYC13 (isolate 1)*
YMM336	*MATα ade2-1 his3-11,15 leu2-3,112 trp1-1 ura3-1 CTF7:ADE2 elg1::KAN URA3:tetO LEU2:tetR-GFP TRP1:PDS1-MYC13 (isolate 1)*
YMM337	*MATα ade2-1 his3-11,15 leu2-3,112 trp1-1 ura3-1 ctf7^eco1-1^:ADE2 elg1::KAN URA3:tetO LEU2:tetR-GFP TRP1:PDS1-MYC13 (isolate 1)*
YMM326	*MATa ade2-1 his3-11,15 leu2-3,112 trp1-1 ura3-1 CTF7:ADE2 elg1::KAN URA3:tetO LEU2:tetR-GFP TRP1:PDS1-MYC13(isolate 2)*
YMM323	*MATa ade2-1 his3-11,15 leu2-3,112 trp1-1 ura3-1 ctf7^eco1-1^:ADE2 elg1::KAN URA3:tetO LEU2:tetR-GFP TRP1:PDS1-MYC13(isolate 2)*
YLL11	*MATa ade2-1 his3-11,15 leu2-3,112 trp1-1 ura3-1 CTF7:ADE2 URA3:tetO LEU2:tetR-GFP TRP1:PDS1-MYC13 (isolate 2)*
YMM282	*MATa ade2-1 his3-11,15 leu2-3,112 trp1-1 ura3-1 CTF7:ADE2 elg1::KAN URA3:tetO LEU2:tetR-GFP TRP1:PDS1-MYC13 (isolate 3)*
YMM309	*MATa ade2-1 his3-11,15 leu2-3,112 trp1-1 ura3-1 CTF7:ADE2 rad24::NAT URA3:tetO LEU2:tetR-GFP TRP1:PDS1-MYC13*
YMM311	*MATa ade2-1 his3-11,15 leu2-3,112 trp1-1 ura3-1 ctf7^eco1-1^:ADE2 rad24::NAT URA3:tetO LEU2:tetR-GFP TRP1:PDS1-MYC13*
YMM313	*MATα ade2-1 his3-11,15 leu2-3,112 trp1-1 ura3-1 CTF7:ADE2 elg1::KAN rad24::NAT URA3:tetO LEU2:tetR-GFP TRP1:PDS1-MYC13*
YMM315	*MATα ade2-1 his3-11,15 leu2-3,112 trp1-1 ura3-1 ctf7^eco1-1^:ADE2 elg1::KAN rad24::NAT URA3:tetO LEU2:tetR-GFP TRP1:PDS1-MYC13 (isolate 1)*
YMM316	*MATα ade2-1 his3-11,15 leu2-3,112 trp1-1 ura3-1 ctf7^eco1-1^:ADE2 elg1::KAN rad24::NAT URA3:tetO LEU2:tetR-GFP TRP1:PDS1-MYC13 (isolate 2)*
YMM360	*MATa ade2-1 his3-11,15 leu2-3,112 trp1-1 ura3-1 ctf7^eco1-1^::ADE2*
YMM403	*MATa ade2-1 his3-11,15 leu2-3,112 trp1-1 ura3-1 CTF7:ADE2*
YMM395**	*MATα ade2-1 his3-11,15 leu2-3,112 trp1-1 ura3*
YMM396**	*MATa ade2-1 his3-11,15 leu2-3,112 trp1-1 ura3 mcd1-1*
YMM397**	*MATα ade2-1 his3-11,15 leu2-3,112 trp1-1 ura3 elg1::KAN*
YMM398**	*MATα ade2-1 his3-11,15 leu2-3,112 trp1-1 ura3 rad24::NAT*
YMM399**	*MATa ade2-1 his3-11,15 leu2-3,112 trp1-1 ura3 mcd1-1 elg1::KAN*
YMM400**	*MATα ade2-1 his3-11,15 leu2-3,112 trp1-1 ura3 mcd1-1 rad24::NAT*
YMM401**	*MATα ade2-1 his3-11,15 leu2-3,112 trp1-1 ura3 elg1::KAN rad24::NAT*
YMM402**	*MATα ade2-1 his3-11,15 leu2-3,112 trp1-1 ura3 mcd1-1 elg1::KAN rad24::NAT*
YBS255*	*MATa ade2-101 his3Δ200 leu2Δ1 trp1Δ63 urs3-52 lys2-801 CTF7::HIS CTF7:LEU2*
YBS514*	*MATa ade2-101 his3Δ200 leu2Δ1 trp1Δ63 urs3-52 lys2-801 CTF7::HIS ctf7-203:LEU2*
YMM173*	*MATa ade2-101 his3Δ200 leu2Δ1 trp1Δ63 urs3-52 lys2-801 CTF7::HIS CTF7:LEU2 elg1::KAN*
YMM236*	*MATa ade2-101 his3Δ200 leu2Δ1 trp1Δ63 urs3-52 lys2-801 CTF7::HIS ctf7-203:LEU2 elg1::KAN*

** strains obtained from backcrossing A364A cells harboring the *mcd1-1* allele twice into W303.

To construct *elg1::KAN* deletion cells, PCR fragments were generated using GTTGCATCTAGATTAAAAGAACGCTACTAA and ATCAGCCATTTTTTTTCAGGTTTTCGC primers with genomic template MBY66 (kindly provided by Dr. Grant Brown) and transformed into a *CEN*-proximal cohesion strain that also contained *CTF7:ADE2*. Proper integration was confirmed by PCR using Primers GCCATCGGTCGTATTGCC and GATTGTCGCACCTGATTGCC. To obtain *ctf7^eco1-1^:ADE3 elg1::KAN* double mutant cells, *CTF7:ADE3 elg1::KAN* cells were mated to *ctf7^eco1-1^:ADE* cells sporulated and the resulting haploid strains scored for single and double mutants. *ctf7^eco1-1^:ADE3 elg1::KAN CEN*-proximal cohesion markers were then backcrossed to *W303* wildtype cells to obtain strains harboring *CTF7:ADE3* and *ctf7^eco1-1^:ADE2 elg1::KAN* devoid of cohesion assay cassette markers.

To obtain *rad24::NAT* deletions cells PCR fragments were generated with primers AACACCTTATTGGACATCTCATCAT and AGAAGTTTTCTGATTGGCCTACTTT with DNA template BY4741 (kindly provided by Dr. Grant Brown). Transformants were confirmed by PCR using primers TCATGGGGTATATCATTGCC.

To obtain *mcd1-1 elg1*, *mcd1-1 rad24::NAT* and *mcd1-1 elg1::KAN rad24::NAT* double and triple mutant strains, *mcd1-1* (kindly provided by Dr. Vincent Guacci) was crossed to *CTF7:ADE elg1::KAN rad24::NAT CEN*-proximal cohesion strain and then sporulated and the appropriate markers identified.

### *ELG1* over expression

Wild type and *ctf7^eco1-1^:ADE3* mutant cells were transformed with pPL448 (2 µm, *URA3*, *GAL1-10 GST-ELG1*) or pPL438 (2 µm, *URA3*, *GAL1-10 GST-CTF18*) kindly provided by Dr. Peter M. Burgers. Log phase cells were normalized and plated on media lacking uracil and containing either glucose or galactose as the carbon source. Plated cells were then maintained at 23°C and growth assessed 4–7 days later.

### Cohesion assay

Cohesion assay procedures were performed as previously described with the following modifications [39]. For α factor pre-arrest trials, log phase cultures were incubated in YPD supplemented with alpha factor (5 µg/ml final concentration) for 3 hours at 23°C. Alpha factor was washed out and cells were rinsed with pre-warmed YPD followed by incubation in YPD supplemented with nocodazole (20 µg/ml final concentration) for 1 hour at 30°C. Samples were collected for Flow Cytometry analysis and paraformaldehyde fixation. Paraformadehyde fixed mitotic cells were probed with 9E10 MYC-directed 9E10 monoclonal antibody (Santa Cruz) to visualize Pds1-MYC and Pds1p positive cells further scored for one or two GFP signals. Cohesion analyses were repeated three times for wildtype cells, two times for *elg1* mutants and four times each for *ctf7^eco1-1^* and *ctf7^eco1-1^ elg1* mutant cells with a minimum of at least 100 cells scored in each trial. DNA content was analyzed by flow cytometry as previously described [25], [28]. For HU pre-arrest trials, log phase wild type and *ctf7^eco1-1^* cells were switched to fresh medium containing 0.2 M hydroxyurea and incubated 2 hours at 23°C followed by 1 hour at 27°. Following washes, cells were then incubated in fresh medium supplemented with nocodozole and maintained at 27°C for 3 hours. Cell aliquots were then analyzed for DNA content, Pds1p staining and Pds1p positive cells further scored for the number of GFP signals. This cohesion analysis was repeated two times with a minimum of 100 cells counted per trial for each strain. Wildtype cells and additional isolates of *elg1* mutant strains were assayed by incubating log phase cells in either hydroxyurea or nocodazole for 3 hours at 23°C and similarly processed for DNA content, Pds1p staining and GFP disposition. These trials were repeated two times with at least 100 cells collected per trial per strain.

## Supporting Information

Figure S1 ELG1 deletion also suppresses ctf7-203 mutant cell conditional growth. 10-fold serial dilutions of wildtype, ctf7 and elg1 single mutant strains compared to ctf7 elg1 double mutant strains. Colony growth on rich medium plates maintained at 23°, 30° and 37° for 7 days are shown.
(2.95 MB TIF)

Figure S2 Elg1p function in sister chromatid cohesion. A) Micrographs of wild type and elg1 mutant cells arrested in G1 (α factor) or early S phase (HU). Cell morphology (DIC) and chromosome disposition (GFP) reveal cells that contain a single GFP focus. B) Micrographs of wild type and elg1 single mutant strains arrested pre-anaphase in which sister chromatid loci (GFP) and Pds1p (Pds1p) are visualized within the DNA mass (DAPI). C) Quantification of cohesion defects exhibited by wild type and elg1 single mutant strains arrested prior to anaphase. D) DNA content of wild type and elg1 single mutant strains during log phase growth (Log), synchronized in early S phase (HU) or pre-anaphase (NZ) at 23°C.
(0.83 MB TIF)
