# Supplementary figures and images for: The Elg1-RFC Clamp-Loading Complex Performs a Role in Sister Chromatid Cohesion
Source: PLoS One. 2009 Mar 5;4(3):e4707. doi: 10.1371/journal.pone.0004707 (PMC2650802; doi:10.1371/journal.pone.0004707)

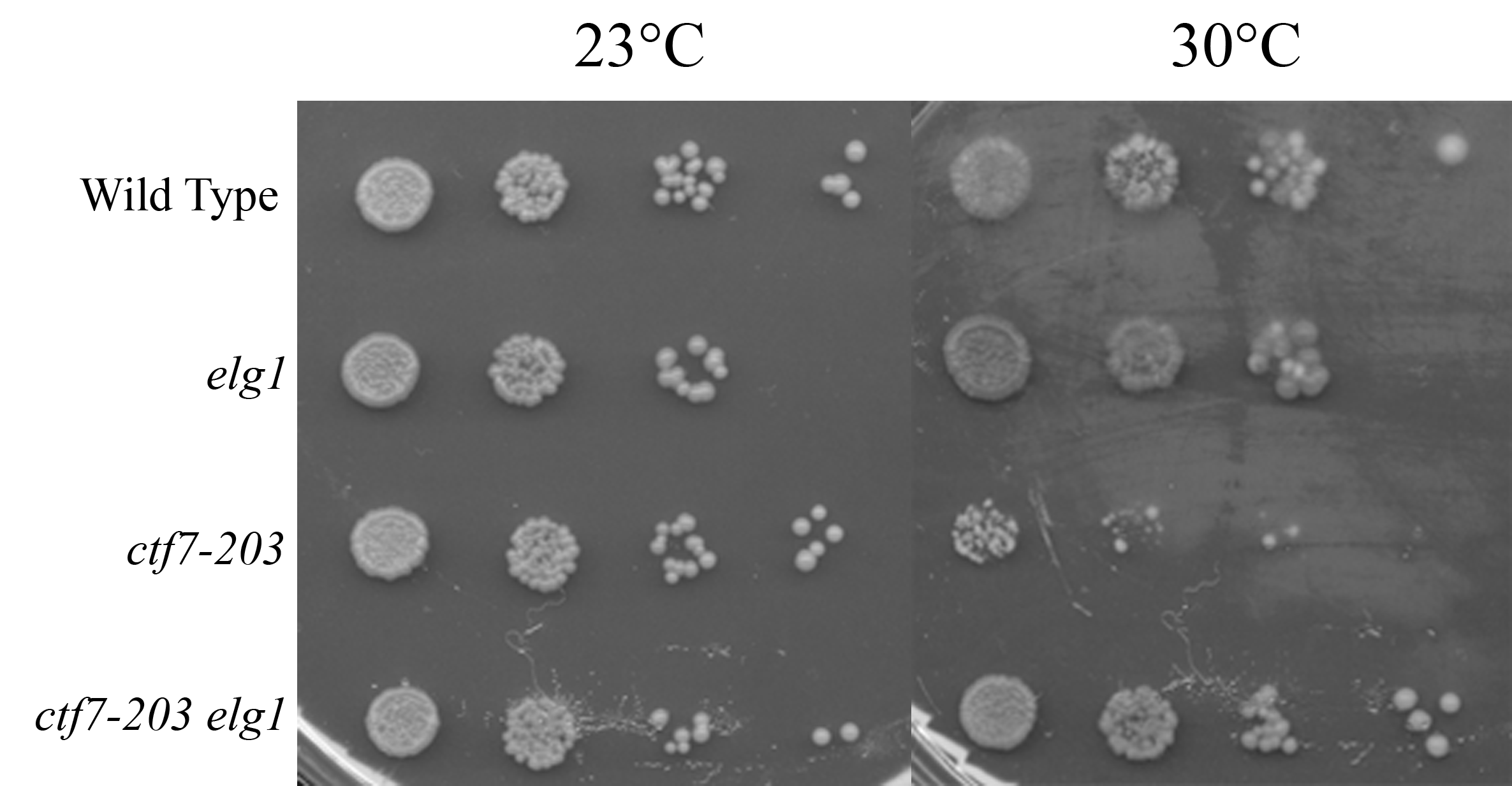

Supplement: Figure S1 — ELG1 deletion also suppresses ctf7-203 mutant cell conditional growth. 10-fold serial dilutions of wildtype, ctf7 and elg1 single mutant strains compared to ctf7 elg1 double mutant strains. Colony growth on rich medium plates maintained at 23°, 30° and 37° for 7 days are shown. (2.95 MB TIF) [file pone.0004707.s001.tif]

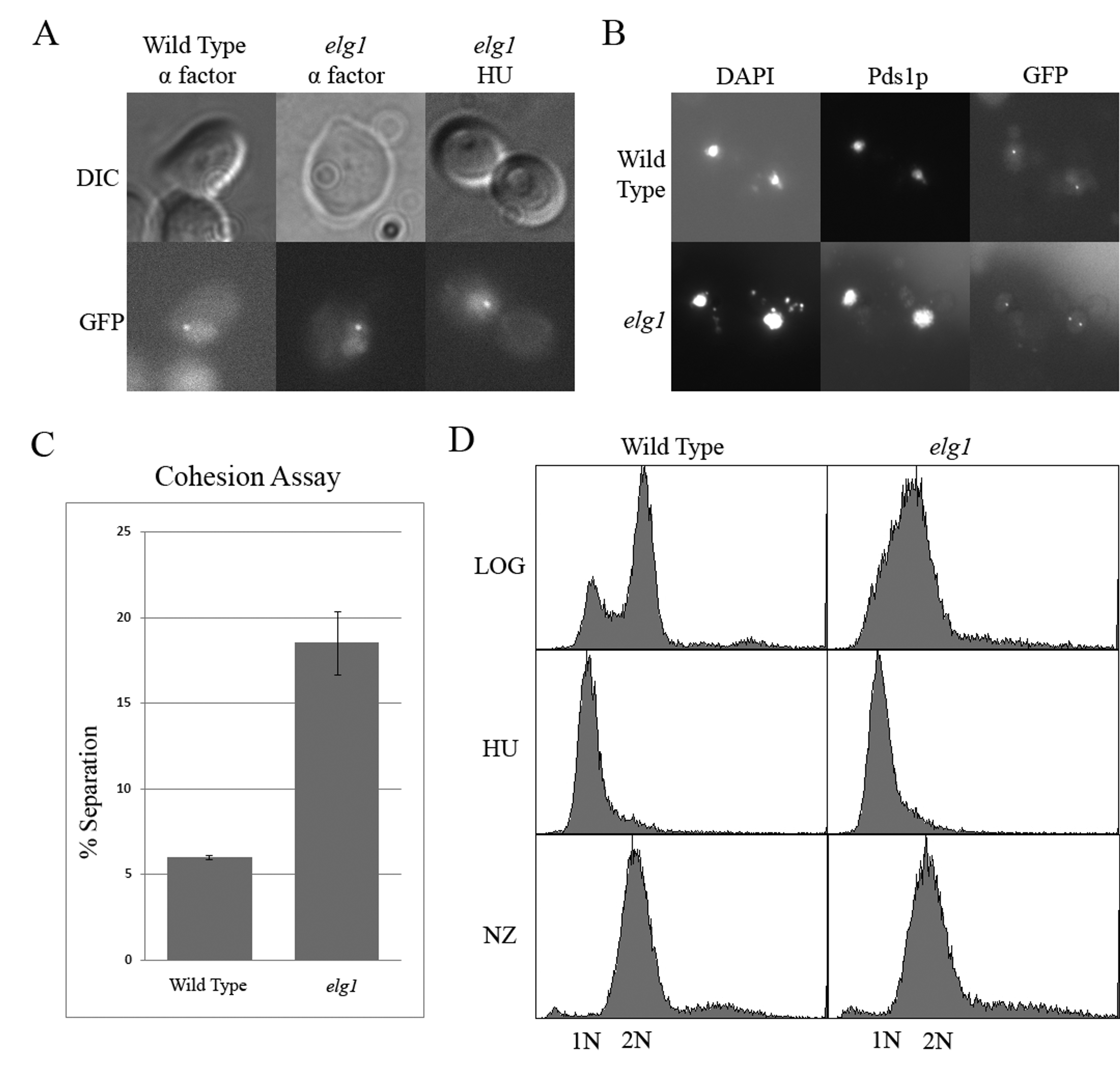

Supplement: Figure S2 — Elg1p function in sister chromatid cohesion. A) Micrographs of wild type and elg1 mutant cells arrested in G1 (α factor) or early S phase (HU). Cell morphology (DIC) and chromosome disposition (GFP) reveal cells that contain a single GFP focus. B) Micrographs of wild type and elg1 single mutant strains arrested pre-anaphase in which sister chromatid loci (GFP) and Pds1p (Pds1p) are visualized within the DNA mass (DAPI). C) Quantification of cohesion defects exhibited by wild type and elg1 single mutant strains arrested prior to anaphase. D) DNA content of wild type and elg1 single mutant strains during log phase growth (Log), synchronized in early S phase (HU) or pre-anaphase (NZ) at 23°C. (0.83 MB TIF) [file pone.0004707.s002.tif]
